# Ultra-Thin ReS_2_ Nanosheets Grown on Carbon Black for Advanced Lithium-Ion Battery Anodes

**DOI:** 10.3390/ma12091563

**Published:** 2019-05-13

**Authors:** Yaping Yan, Kyeong-Youn Song, Minwoo Cho, Tae Hoon Lee, Chiwon Kang, Hoo-Jeong Lee

**Affiliations:** 1 School of Advanced Materials Science and Engineering, Sungkyunkwan University (SKKU), Suwon 16419, Korea; yaping1006@skku.edu (Y.Y.); cll7020@skku.edu (M.C.); 2 Department of Physics and Institute of Basic Science, Sungkyunkwan University, 2066, Seobu-ro, Jangan-gu, Suwon 16419, Gyeonggi-do, Korea; 3 SKKU Advanced Institute of Nano Technology (SAINT), Sungkyunkwan University, Suwon 16419, Korea; echirolles@skku.edu; 4 Center for Integrated Nanostructure Physics (CINAP), Institute for Basic Science (IBS), Suwon 16419, Korea; hooni0629@skku.edu; 5 Department of Energy Science, Sungkyunkwan University (SKKU), Suwon 16419, Korea

**Keywords:** ultra-thin ReS_2_ nanosheets, carbon black (CB), lithium-ion battery (LIB)

## Abstract

ReS_2_ nanosheets are grown on the surface of carbon black (CB) via an efficient hydrothermal method. We confirmed the ultra-thin ReS_2_ nanosheets with ≈1–4 layers on the surface of the CB (ReS_2_@CB) by using analytical techniques of field emission scanning electron microscopy (FESEM) and high-resolution transmission electron microscopy (HRTEM). The ReS_2_@CB nanocomposite showed high specific capacities of 760, 667, 600, 525, and 473 mAh/g at the current densities of 0.1 (0.23 C), 0.2 (0.46 C), 0.3 (0.7 C), 0.5 (1.15 C) and 1.0 A/g (2.3 C), respectively, in conjunction with its excellent cycling performance (432 mAh/g at 2.3 C; 91.4% capacity retention) after 100 cycles. Such LIB performance is greatly higher than pure CB and ReS_2_ powder samples. These results could be due to the following reasons: (1) the low-cost CB serves as a supporter enabling the formation of ≈1–4 layered nanosheets of ReS_2_, thus avoiding its agglomeration; (2) the CB enhances the electrical conductivity of the ReS_2_@CB nanocomposite; (3) the ultra-thin (1–4 layers) ReS_2_ nanosheets with imperfect structure can function as increasing the number of active sites for reaction of Li^+^ ions with electrolytes. The outstanding performance and unique structural characteristics of the ReS_2_@CB anodes make them promising candidates for the ever-increasing development of advanced LIBs.

## 1. Introduction

Li-ion batteries (LIBs), as an advanced energy storage system, have been employed as a main power source of most commercially available electronics and currently emerging electric vehicles due to their high energy density, low self-discharge, zero to low memory effect, quick charging, and longer lifespan [1,2,3,4,5]. The profound investigation of novel electrode materials has been one of core research areas to meet the ever-demanding need for high-performance LIBs. Transition metal sulfides (TMSs) have received great attention as a highly efficient anode material owing to their multi-faceted traits including a relatively small volume change and superior reversibility during charge–discharge cycling, and higher electrical conductivity because of the weaker M–S bonds relative to its M–O counterparts of transition metal oxides (TMOs) [6] with layered structures (e.g., MoS_2_ and WS_2_) [7]. TMSs possess their weaker van der Waals interaction between S–S interlayers, which enables an excellent buffering against mechanical stress induced by volume change during the charge–discharge cycling process compared with other TMO-based anode materials [8]. The above-mentioned advantages also allow the TMDs to hold great potential as lithium-ion capacitors (LIC) [9,10,11]. 

Among the TMS-based anodes, ReS_2_ has recently been investigated for its enhanced Li^+^ ion transport kinetics for practical LIB applications because of its unique properties of a weak S–S interlayer binding strength and a large interlayer distance [12]. Nevertheless, the inferior LIB performance of ReS_2_ at the higher current density has hindered its practical application. To overcome this limitation, one of the most legitimate strategies is to synthesize nanocomposites of ReS_2_ with a variety of morphologies (nanowalls, nanoflowers, and nanosheets) combined with the nanocarbon-based materials (3D graphene foam, N-doped carbon nanofiber-based paper, and CNTs [12,13,14,15,16,17]). In these nanocomposites, nanocarbon can buffer against mechanical stress caused by volume variation, enhance an electrical and ionic conductivity, and enable the microstructural and morphological change of ReS_2_. However, what has been overlooked until recently is the design and fabrication a ReS_2_-based anode material for exceptional LIB performance at high current density.

Herein, to address this issue, we, for the first time, fabricated ReS_2_ nanosheets with ultra-thin thickness comprising ~1–4 layers grown on CB through a hydrothermal synthesis method. The synthesized nanocomposite has unique properties to achieve high Li^+^ ion transport kinetics, especially at high current density, including [17,18]: (1) excellent combination between ReS_2_ and CB; (2) the ReS_2_ nanosheets’ sufficient space for reaction with electrolytes; (3) small size (≈20 nm), ultra-thin thickness (≈1–4 layers) and defect structure of the ReS_2_ nanosheets to accelerate the Li^+^ ion transport and buffer against mechanical stress induced by volume variation during the cycling process. With these unique physico-chemical characteristics, as-synthesized ReS_2_@CB nanocomposites shows high specific capacities of 760, 667, 600, 525, and 473 mAh/g at current densities of 0.1 (0.23 C), 0.2 (0.46 C), 0.3 (0.7 C), 0.5 (1.15 C), and 1.0 A/g (2.3 C), respectively, along with its excellent capacity retention of 91.4% with a high capacity of 432 mAh/g at 1.0 A/g (2.3 C) after 100 cycles. Such excellent rate capability and superior cycling stability could open up new, challenging prospects in this ever-increasing development of TMS-based anode materials for advanced LIBs.

## 2. Materials and Methods

### 2.1. Synthesis of ReS_2_@CB and ReS_2_ Powder

**Figure 1:**
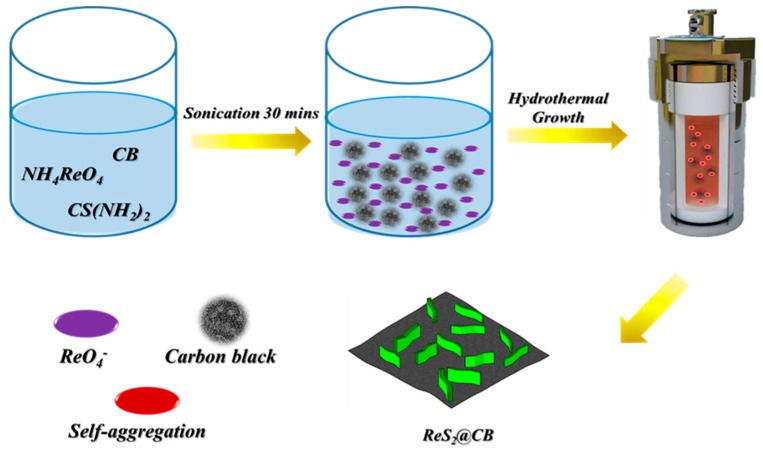
The schematic diagram illustrating each synthesis process for ReS_2_@CB structure.

We fabricated ReS_2_@CB through a simple hydrothermal synthesis process [17]. In typical synthesis, 0.3 g of NH_4_ReO_4_, 0.8 g of CS(NH_2_)_2_, 0.35 g HONH_3_Cl, and 20 mg of CB were dissolved into 30 mL of deionized (DI) water and then sonicated for 30 minutes to make a homogeneous solution using a sonicator (UC-10, Lab Companion, Seoul, Korea). Afterwards, the solution was transferred to a 50 mL Teflon-lined stainless-steel autoclave in air atmosphere; subsequently, the autoclave was pressurized at 180 °C for 30 hours in electrical furnace (C-22P, Hantech, Gunpo-si, Korea). After synthesis, we purified the as-synthesized ReS_2_@CB particulate sample via centrifugation at 7000 rpm (CF-10, Daihan Scientific, Seoul, Korea) with ethanol and deionized (DI) water for three times, respectively. For comparison, ReS_2_ powder was prepared with the same process without adding CB. Finally, the purified ReS_2_@CB and ReS_2_ were dried at 80 °C overnight in a drying oven. Figure 1 schematically depicts each synthesis procedure for the ReS_2_@CB structure.

### 2.2. Structural Analysis of ReS_2_@CB, ReS_2_, and CB

The crystal structures of the as-synthesized ReS_2_@CB and ReS_2_ samples were identified using an X-ray diffractometer (XRD) (Miniflex 600, Rigaku, Tokyo, Japan). The morphological and microstructural features of the samples were analysed using field emission scanning electron microscopy (FESEM) (JSM-6701F, JEOL, Tokyo, Japan) and high-resolution transmission electron microscope (HR-TEM) (JEM-2100F, JEOL, Tokyo, Japan). The chemical composition, electronic states, and purity of the ReS_2_@CB and ReS_2_ samples were investigated using X-ray photoelectron spectroscopy (XPS) (MultiLab 2000 system, Thermo Scientific, Waltham, MA, USA). The specific surface area and pore volume of the ReS_2_@CB and CB were measured using Brunauer–Emmett–Teller (BET) equipment (Micromeritics, Norcross, GA, USA; ASAP2020). We used Horvath–Kawazoe (HK) and Barrett–Joyner–Halenda (BJH) methods to acquire micropore and meso-/macro-pore size distributions, respectively.

### 2.3. Lithium Ion Battery Performance of ReS_2_@CB, ReS_2_ Powder, and CB Anode Structures

The electrode slurry was fabricated by mixing active materials (ReS_2_@CB and ReS_2_ powder) (80 wt%), Carbon Black (CB) (Thermo Fisher Scientific, Waltham, MA, USA) (10 wt%) as a conducting agent, and polyvinylidene difluoride (PVDF) (Sigma-Aldrich, Saint Louis, MO, USA) (10 wt%) as a binder in the solvent of *n*-methyl-2-pyrrolidone (Sigma-Aldrich, Saint Louis, MO, USA). For the CB anode sample, the weight ratio of CB and PVDF was 8:2. Subsequently, the slurry was uniformly pasted onto a copper foil as a current collector to produce the anode samples, which were then dried at 80 °C in a drying oven overnight. In a dry room, the coin-type of cells were assembled with the ReS_2_@CB, ReS_2_ powder, and CB as working electrodes; a monolayer polypropylene (PP) film (Celgard, 2400, Charlotte, NC, USA) as a separator; 1 M LiPF_6_ in ethylene carbonate (EC):dimethyl carbonate (DMC) (volume ratio of 1:1) as an electrolyte (Soulbrain, Seongnam-si, Korea); and lithium foil as counter and reference electrodes. With these assembled cells, the galvanostatic charge and discharge cycling test was carried out within a voltage window of 0.01 to 3 V versus Li^+^/Li by using a multi-channel battery testing unit (Maccor, Series 4000, Tulsa, OK, USA). The cyclic voltammetry (CV) for the ReS_2_@CB anode sample was conducted using a multi-channel potentiostat (VMP3, Bio Logic, Seyssinet-Pariset, France) in the same voltage window at scan rates of 0.1, 0.2, 0.4, 0.6, 0.8, 1.0 mV/s. Electrochemical impedance spectroscopy (EIS) was performed on the electrochemical workstation (Bio Logic Science instrument-VSP) in the frequency range from 100 kHz to 0.1 Hz with an alternative current (AC) voltage amplitude of 5.0 mV. 

## 3. Results and Discussion

### 3.1. Schematic Synthesis Process of ReS_2_@CB and Morphological Properties of ReS_2_@CB, ReS_2_ Powder, and CB

**Figure 2:**
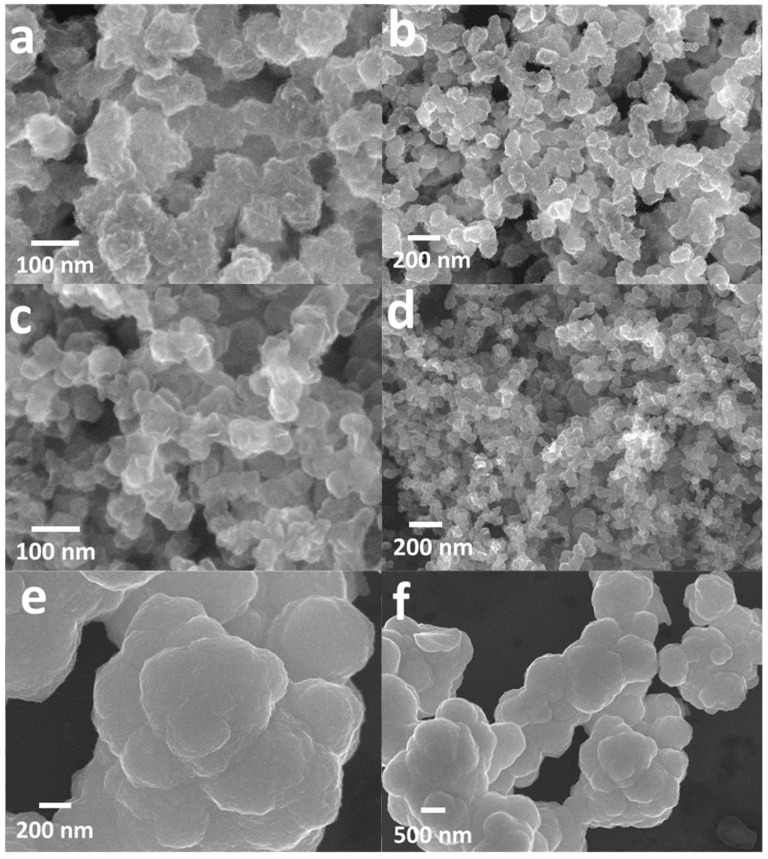
SEM images of the (**a**,**b**) ReS_2_@CB, (**c**,**d**) pure CB and (**e**,**f**) ReS_2_ powder.

As shown in the Figure 1, to have a homogeneous state, the solution went through ultrasonic treatment for 30 minutes. Through a hydrothermal synthetic process with the homogeneous solution, we fabricated the ultra-thin ReS_2_ nanaosheets on CB. The morphology of the as-synthesized ReS_2_@CB structure was analyzed using the FESEM images. Figure 2a,b show uniformly distributed ultra-thin ReS_2_ structures grown on the surface of CB using the hydrothermal method. The as-purchased CB exhibited its small size (<100 nm) with a smooth surface (see Figure 2c,d) and the ReS_2_ powder showed highly agglomerated characteristics (see Figure 2e,f), thus indicating the critical effect of the CB template on the morphology of ReS_2_. These CB sheets facilitated the electrical conductivity of the ReS_2_ and served as a buffering agent against mechanical stress induced by the large volume variation of the ReS_2_ during cycling [19]. The ReS_2_ on the CB could also enhance the active surface area utilization compare with the pure ReS_2_ powder showing its aggregation tendency, which is advantageous for accommodating a large amount of Li^+^ ions into the host structure of the ReS_2_ [20]. 

### 3.2. Structural Property of ReS_2_@CB, ReS_2_ Powder, and CB

**Figure 3:**
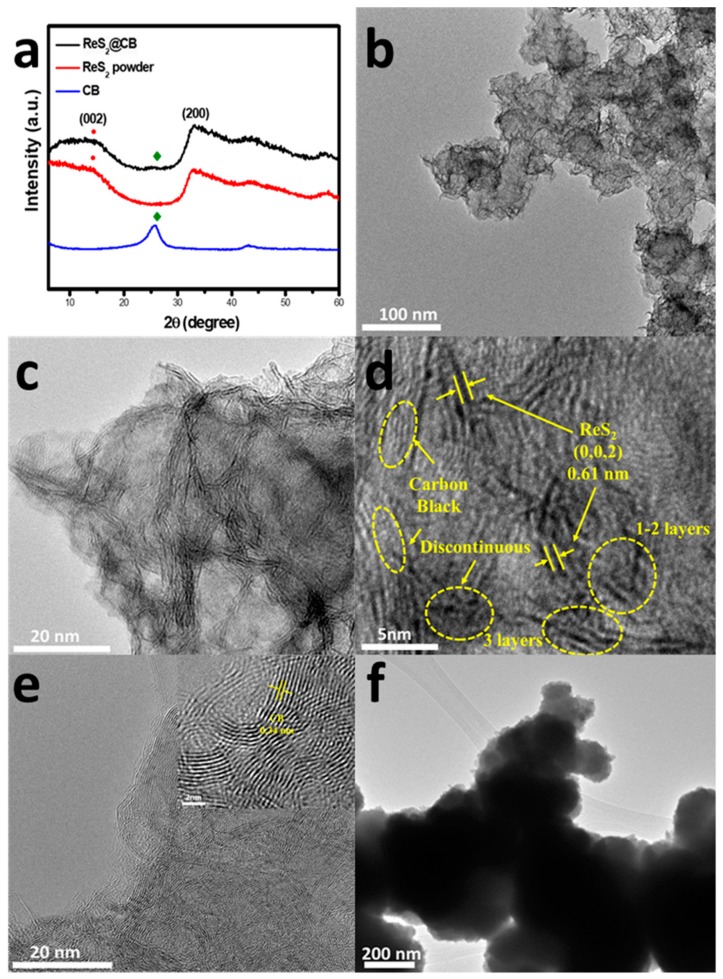
(**a**) XRD pattern of the ReS_2_@CB, ReS_2_ powder, and CB; (**b**,**c**) TEM images of the microstructures of the ReS_2_@CB; (**d**,**e**) HR-TEM images of the ReS_2_@CB and pure CB; and (**f**) TEM image of the ReS_2_ powder.

Figure 3a demonstrates the XRD pattern showing the presence of the ReS_2_ phase with characteristic peaks (JCPDS No. 89-0341) [13], thus proving the ReS_2_ was successfully grown on CB through the hydrothermal process without any side products. The broad peak appearing at 14.5° corresponds to the ReS_2_ (002) plane, suggesting its low crystallinity [21]. Consistent with the aforementioned FESEM analysis, TEM images shown in Figure 3 demonstrate the well-distributed ReS_2_ nanoparticles on CB. Random space between the interlayers of the ReS_2_ was produced in the composite as shown in Figure 3b. Interplanar distances in the box were measured to be ≈0.61 nm, corresponding to the (002) crystalline plane of ReS_2_, which is agreement with the standard JCPDS card (No. 89-0341). The d-spacing (0.61 nm) for the (002) plane of the ReS_2_@CB sample also proved the vertical orientation of the ReS_2_ nanosheets on CB, which was consistent with the FESEM images shown in Figure 2a. According to the HR-TEM images of the nanocomposite, we could identify the few-layered (e.g., ~1–4 layers) nanosheet property. We also observed the discontinuous arrays of the ReS_2_ layers. These defect structures may be beneficial for realizing a high capacity in the LIB performance as they can provide more active sites for Li^+^ ion accommodation, thus enhancing LIB performance [22]. Alternatively, the CB showed an interlayer spacing of ~0.34 nm measured using the HR-TEM image. We found the BET surface area of the ReS_2_@CB was 39.4 m^2^/g according to the N_2_ adsorption–desorption isotherms (see Figure S1). 

### 3.3. X-ray Photoelectron Spectroscopy (XPS) Analysis of ReS_2_@CB

**Figure 4:**
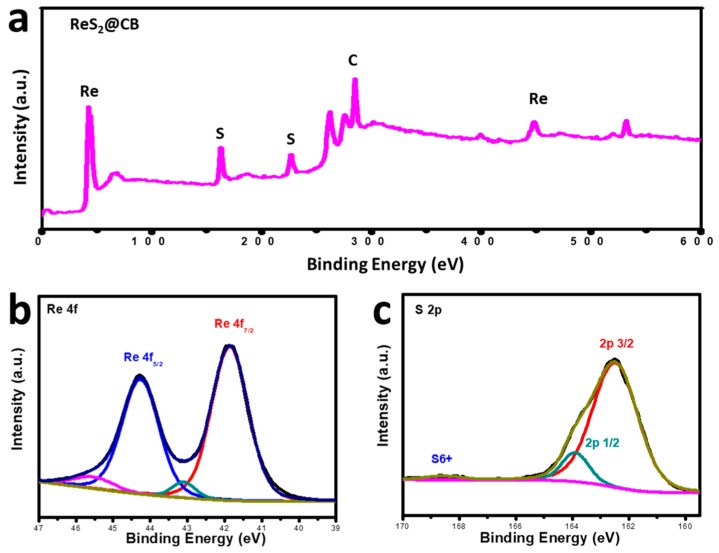
XPS spectra of the ReS_2_@CB: survey spectra (**a**), and high-resolution spectra of Re 4f (**b**) and S 2p (**c**).

We also investigated the bonding interactions between different components of the ReS_2_@CB nanocomposite sample using XPS. Figure 4a shows a full scan of the composite, proving the presence of the Re, S, and C elements. Characteristic peaks arising from Re 4f_7/2_ and Re 4f_6/2_ orbitals are located at 41.9 and 44.2 eV [14], respectively, indicating the dominance of Re(V) in the ReS_2_ product (Figure 4b). In Figure 4c, the S 2p scan also displayed two main peaks at 162.5 and 163.9 eV [23,24] corresponding to the 2p_3/2_ and 2p_1/2_, respectively. The peak at 168.5 eV corresponds to the 6+ oxidation state of sulfur [25,26,27]. 

### 3.4. Electrochemical Performance of ReS_2_@CB, ReS_2_ Powder, and CB

**Figure 5:**
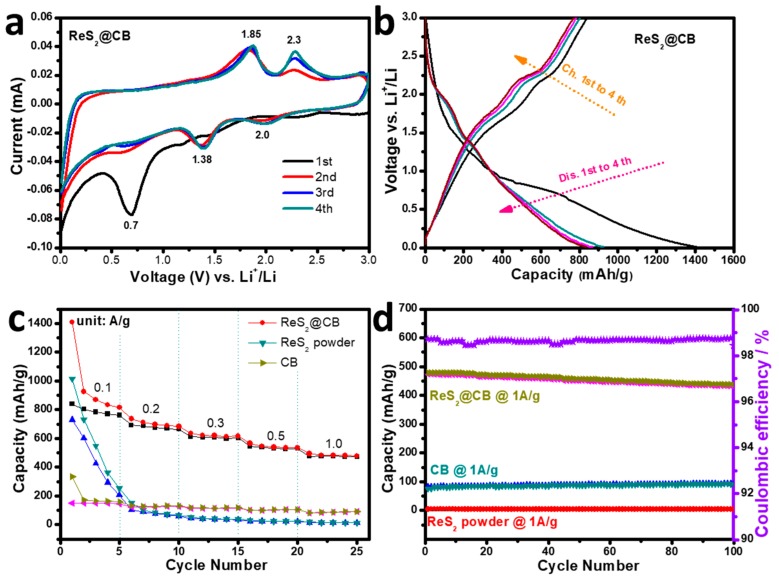
(**a**) CV curve of the ReS_2_@CB, (**b**) galvanostatic charge/discharge (GCD) process of the ReS_2_@CB in the initial four cycles; (**c**) rate capability of the as-prepared samples at different current densities; and (**d**) cycling performance of each sample at 1 A/g and the corresponding coulombic efficiency of the ReS_2_@CB.

The ReS_2_@CB nanocomposite anode was used for a coin-type cell to evaluate its LIB performance (Figure 5). We used cyclic voltammetry (CV) with the anode at a scan rate of 0.1 mV/s in the voltage range of 0.01 to 3.0 V (Figure 5a). The curves were well superimposed and reversible, which meant a good cycling stability of the anode. The strong peak appearing at ~1.2 V during cathodic sweep due to the formation of Li_x_ReS_2_ (where 0 < x < 1) [17], which was caused by the intercalation of Li^+^ ion into ReS_2_@CB [1,10,11]. The transformation occurs at the peak of ~0.7 V along with solid electrolyte interphase (SEI) formation and the complete reduction from Re^4+^ to Re^0^ in the Re region embedded in a Li_2_S phase [28,29]. During anodic sweep, the peaks that occurred at ~1.85 and 2.3 V were closely related to the oxidation reaction of Li_2_S [17,30]. 

All the curves were well-superimposed, thus showing the reversible conversion reaction between ReS_2_ and Li. Figure 5b shows the galvanostatic discharge/charge profiles of ReS_2_@CB in the initial four cycles. During the first discharge, the significant voltage plateaus at around 0.7 V are observed, which corresponds well to the cathodic peak in the CV curves and in the subsequent charge, where the clear plateau at ~2.3 V agrees well with the anodic peak during the CV scan, which is consistent with the previous reports of ReS_2_-based anodes [14]. ReS_2_ is a semiconducting material with a band gap of 1.35 eV and suffers from the volume expansion/contraction to cause detrimental pulverization problems. To overcome this limitation, CB is incorporated with ReS_2_ to facilitate electrical conductivity and guard the volume variation during charge and discharge cycles [31]. This principle is proven by the higher LIB performance of ReS_2_@CB compared to those of pure CB and ReS_2_ samples. For the ReS_2_@CB, the specific capacity decreased from 840 mAh/g for the first cycle to 761 mAh/g for the fifth cycle, whereas coulombic efficiency increases from 60 to 93%, meaning enhanced capacity reversibility with cycling. The initial capacity loss was mainly attributed to irreversible processes such as the inevitable formation of a solid electrolyte interphase (SEI) film on the electrode surface and side-reactions between Li^+^ ion and the active material [17,28]. The ReS_2_@CB nanocomposite shows a high specific capacity of 760, 667, 600, 525, and 473 mAh/g at the current densities of 0.1, 0.2, 0.3, 0.5, and 1.0 A/g, respectively; these values were higher than those of CB (82 mAh/g @ 1A/g) and ReS_2_ powder (13 mAh/g @ 1A/g) as shown in Figure 5c. We performed cycling stability test up to 100 cycles for the three different samples of ReS_2_@CB, CB, and ReS_2_ powder at 1 A/g. Such excellent cycling stability with a high specific capacity of 432 mAh/g for ReS_2_@CB anode sample was closely attributed to the CB, as well as the vertical orientation with a defect property which offered high electrical conducting pathways and accelerated Li^+^ ion diffusion rate, thus improving the kinetic efficiency of Li-storage [32]. The coulombic efficiency reached 98.8% over the entire 100 cycles. In contrast, the pure ReS_2_ anode sample shows a negligible capacity of ~3 mAh/g at 1 A/g, confirming the crucial role of CB to enhance LIB performance. Furthermore, the pure CB anode sample exhibited a specific capacity of ~92 mAh/g after 100 cycles. According to these results, we can interpret that the higher specific capacities stemmed from the crucial role of the incorporated CB to promote electrical conducting pathways for ReS_2_, buffer against a large volume change of ReS_2_ during cycling and enable the ultrathin thickness of the ReS_2_@CB to expose more active sites, thereby improving Li^+^ ion kinetics.

**Figure 6:**
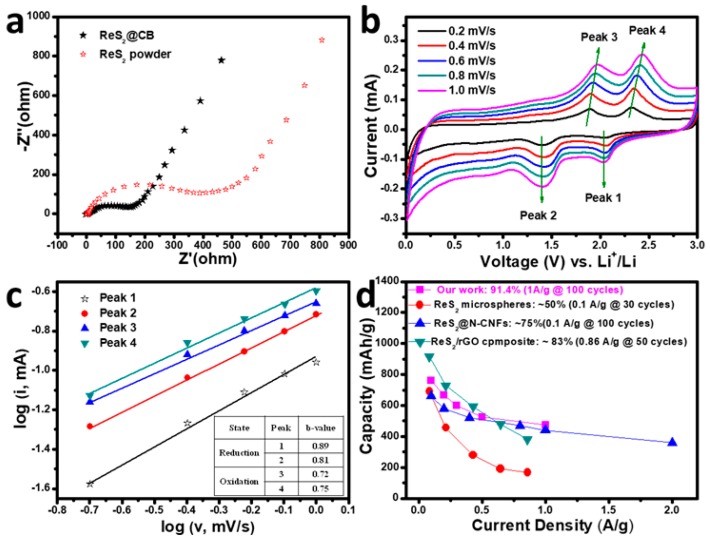
(**a**) EIS data of the ReS_2_@CB and ReS_2_ powder samples, (**b**) CV curves of the ReS_2_@CB electrode at the different scan rates, (**c**) correlation between logarithmic peak current (cathodic and anodic peaks) versus logarithmic voltage scan rate, and (**d**) comparison of capacity at the different current densities for the ReS_2_@CB with those of reported ReS_2_-based composite anodes.

Electrochemical impedance spectroscopy (EIS) measurement were performed with our samples to evaluate the charge transfer resistance of the ReS_2_@CB and ReS_2_ powder samples (see Figure 6a). Significantly, the diameter of the semicircle for the ReS_2_@CB (139 Ω) in the high-medium frequency region was smaller than that of the ReS_2_ powder (460 Ω), which indicates that ReS_2_@CB possessed the lower contact and charge-transfer resistances (R_ct_) and the slope of the ReS_2_@CB in the low frequency region is also higher than the ReS_2_ powder. These EIS results prove that the ReS_2_@CB architecture showed a high electrical conductivity of the overall electrode and enhanced the Li^+^ ion diffusion of the ReS_2_ anode during cycling process. 

To understand the deep insight into the improved electrochemical performance, we investigated the capacitive effect of the battery system by testing CV curves of the ReS_2_@CB at the different sweep rates from 0.2 to 1.0 mV/s (Figure 6b). The capacitive contribution to the battery system was calculated according to the following equations [33,34]:

(1) $$i=av^{b}\;$$

(2) logi=b×logv+loga

where *i* and *v* are the current density and scan rate, respectively, and *a* and *b* are adjustable parameters. In general, when the *b*-value is close to 1, the system was mainly controlled by surface-controlled process or capacitance behavior. In contrast, when the *b*-value was close to 0.5, the system was mainly dominated by a diffusion-controlled process [34]. As displayed in the inset table in Figure 6c, the higher *b* values of the cathodic an d anodic peaks (0.89 and 0.72 for peak 1 and 3, respectively) suggest the more favored capacitive kinetics of the ReS_2_@CB electrode, which could be due to the ultra-thinness and imperfect crystallinity of the ReS_2_@CB. Compared with the published values, our data show the superior specific capacity and cycling performance at 1 A/g as shown in Figure 6d [13,15,16].

## 4. Conclusions

In summary, we synthesized the ReS_2_@CB anode structure with ultra-thinness (~1–4 layers) and low-degree crystallinity through the hydrothermal process. When employed in the LIB performance, the synthesized ReS_2_@CB (473 mAh/g @ 1 A/g) showed a higher rate capability and higher capacity than the CB (82 mAh/g @ 1 A/g) and ReS_2_ powder (13 mAh/g @ 1 A/g), as well as an excellent cycling retention of 91.4% (@ 1 A/g), even after 100 cycles. The improved electrochemical performance of our ReS_2_@CB nanocomposite anode was closely attributed to the decisive roles of CB on the enhanced electrical conductivity of ReS_2_ and the ultrathin nanosheets with a defect structure to facilitate the Li^+^ ion diffusion rate, thus improving the kinetic efficiency of the redox reaction during the cycling process. Furthermore, with these unique physico-chemical properties, we confirmed that the investigation on the ReS_2_@CB anode would open up a new avenue of pursuing high-performance TMS-based anodes for advanced LIBs.

## Supplementary Materials

The following are available online at https://www.mdpi.com/1996-1944/12/9/1563/s1, Figure S1: N_2_ adsorption–desorption isotherms of (**a**) ReS_2_@CB and (**b**) CB.
