# Supplementary material for: Ultra-Thin ReS2 Nanosheets Grown on Carbon Black for Advanced Lithium-Ion Battery Anodes
Source: Materials (Basel). 2019 May 13;12(9):1563. doi: 10.3390/ma12091563 (PMC6539948; doi:10.3390/ma12091563)
Supplement: Supplementary file 1 [file materials-12-01563-s001.pdf]

Supplementary Information

Ultra-Thin ReS_2 Nanosheets grown on Carbon Black for Advanced Lithium-Ion Battery Anode

Yaping Yan [1,2,†], Kyeong-Youn Song [3,†], Minwoo Cho [1], Tae Hoon Lee [4,5], Chiwon Kang [1,*] and Hoo-Jeong Lee [1,3,*]

[1] School of Advanced Materials Science and Engineering, Sungkyunkwan University (SKKU), Suwon 16419, Republic of Korea; hlee@skku.edu; cll7020@skku.edu

[2] Department of Physics and Institute of Basic Science, Sungkyunkwan University, 2066, Seobu-ro, Jangan-gu, Suwon 16419, Gyeonggi-do, Republic of Korea; yaping1006@skku.edu

[3] SKKU Advanced Institute of Nano Technology (SAINT), Sungkyunkwan University, Suwon 16419, Republic of Korea; echirolles@skku.edu

[4] Center for Integrated Nanostructure Physics (CINAP), Institute for Basic Science (IBS), Suwon 16419, Republic of Korea; hooni0629@skku.edu

[5] Department of Energy Science, Sungkyunkwan University (SKKU), Suwon 16419, Republic of Korea

* Correspondence: chiwonkang@skku.edu (C.K); hlee@skku.edu (H.-J.L.); Tel.: +82-31-299-4735 (C.K.); +82-31-290-7365 (H.-J.L.)

† These co-first authors contributed equally to this work

Received: 30 March 2019; Accepted: 9 May 2019; Published: date

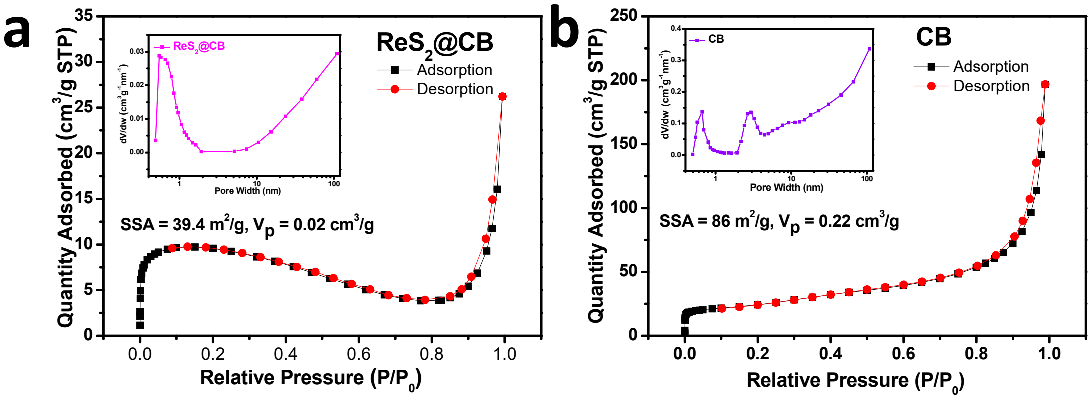

Figure S1. N_2 adsorption–desorption isotherms of **(a)** ReS_2@CB and **(b)** CB. The black squares corresponds to N_2 adsorption, whereas the red circles N_2 desorption, the inserted figures show the pore size distribution of the ReS_2@CB and CB, respectively.
